# Supplementary material for: OpenMM 7: Rapid development of high performance algorithms for molecular dynamics
Source: PLoS Comput Biol. 2017 Jul 26;13(7):e1005659. doi: 10.1371/journal.pcbi.1005659 (PMC5549999; doi:10.1371/journal.pcbi.1005659)
Supplement: S1 Source Code — (ZIP) [file pcbi.1005659.s002.zip › OpenMM-7.0.1-Source/docs/api-python/generated/simtk.openmm.app.amberprmtopfile.AmberPrmtopFile.html]

AmberPrmtopFile — OpenMM 7.0.1.dev-5e86c4f documentation


# AmberPrmtopFile¶

*class* `simtk.openmm.app.amberprmtopfile.``AmberPrmtopFile`(*file*)¶
:   AmberPrmtopFile parses an AMBER prmtop file and constructs a Topology and (optionally) an OpenMM System from it.

    `__init__`(*file*)¶
    :   Load a prmtop file.

    Methods

    |  |  |
    | --- | --- |
    | `__init__`(file) | Load a prmtop file. |
    | `createSystem`([nonbondedMethod, ...]) | Construct an OpenMM System representing the topology described by this prmtop file. |

    `__delattr__`¶
    :   x.\_\_delattr\_\_(‘name’) <==> del x.name

    `__format__`()¶
    :   default object formatter

    `__getattribute__`¶
    :   x.\_\_getattribute\_\_(‘name’) <==> x.name

    `__hash__`¶

    `__reduce__`()¶
    :   helper for pickle

    `__reduce_ex__`()¶
    :   helper for pickle

    `__repr__`¶

    `__setattr__`¶
    :   x.\_\_setattr\_\_(‘name’, value) <==> x.name = value

    `__sizeof__`() → int¶
    :   size of object in memory, in bytes

    `__str__`¶

    `createSystem`(*nonbondedMethod=NoCutoff*, *nonbondedCutoff=Quantity(value=1.0*, *unit=nanometer)*, *constraints=None*, *rigidWater=True*, *implicitSolvent=None*, *implicitSolventSaltConc=Quantity(value=0.0*, *unit=mole/liter)*, *implicitSolventKappa=None*, *temperature=Quantity(value=298.15*, *unit=kelvin)*, *soluteDielectric=1.0*, *solventDielectric=78.5*, *removeCMMotion=True*, *hydrogenMass=None*, *ewaldErrorTolerance=0.0005*, *switchDistance=Quantity(value=0.0*, *unit=nanometer)*)¶
    :   Construct an OpenMM System representing the topology described by this
        prmtop file.

        |  |  |
        | --- | --- |
        | Parameters: | - **nonbondedMethod** (*object=NoCutoff*) – The method to use for nonbonded interactions. Allowed values are   NoCutoff, CutoffNonPeriodic, CutoffPeriodic, Ewald, or PME. - **nonbondedCutoff** (*distance=1\*nanometer*) – The cutoff distance to use for nonbonded interactions - **constraints** (*object=None*) – Specifies which bonds angles should be implemented with constraints.   Allowed values are None, HBonds, AllBonds, or HAngles. - **rigidWater** (*boolean=True*) – If true, water molecules will be fully rigid regardless of the value   passed for the constraints argument - **implicitSolvent** (*object=None*) – If not None, the implicit solvent model to use. Allowed values are   HCT, OBC1, OBC2, GBn, or GBn2. - **implicitSolventSaltConc** (*float=0.0\*unit.moles/unit.liter*) – The salt concentration for GB calculations (modelled as a debye   screening parameter). It is converted to the debye length (kappa)   using the provided temperature and solventDielectric - **temperature** (*float=300\*kelvin*) – Temperature of the system. Only used to compute the Debye length   from implicitSolventSoltConc - **implicitSolventKappa** (*float units of 1/length*) – If this value is set, implicitSolventSaltConc will be ignored. - **soluteDielectric** (*float=1.0*) – The solute dielectric constant to use in the implicit solvent model. - **solventDielectric** (*float=78.5*) – The solvent dielectric constant to use in the implicit solvent   model. - **removeCMMotion** (*boolean=True*) – If true, a CMMotionRemover will be added to the System - **hydrogenMass** (*mass=None*) – The mass to use for hydrogen atoms bound to heavy atoms. Any mass   added to a hydrogen is subtracted from the heavy atom to keep their   total mass the same. - **ewaldErrorTolerance** (*float=0.0005*) – The error tolerance to use if nonbondedMethod is Ewald or PME. - **switchDistance** (*float=0\*nanometers*) – The distance at which the potential energy switching function is   turned on for Lennard-Jones interactions. If the switchDistance is 0   or evaluates to boolean False, no switching function will be used.   Values greater than nonbondedCutoff or less than 0 raise ValueError |
        | Returns: | the newly created System |
        | Return type: | System |

High performance molecular simulation on GPUs


### Navigation

- Application Layer
  - Loaders and Setup
  - Representation and Manipulation
  - Simulation
  - Reporting Output
  - Extras
- Library Layer

©2015, Stanford University and the Authors.
|
Powered by Sphinx 1.3.1
& Alabaster 0.7.7
|
Page source
